# The Effects of the Ecological Conservation Redline in China: A Case Study in Anji County

**DOI:** 10.3390/ijerph19137701

**Published:** 2022-06-23

**Authors:** Chao Zhang, Dayi Lin, Lixia Wang, Haiguang Hao, Yuanyuan Li

**Affiliations:** 1 State Key Laboratory of Environmental Criteria and Risk Assessment, Chinese Research Academy of Environmental Sciences, Beijing 100012, China; zhangchao_sd@126.com (C.Z.); lindyhit@foxmail.com (D.L.); 13511038653@126.com (Y.L.); 2 Satellite Application Center for Ecology and Environment, Ministry of Ecological and Environment of the People’s Republic of China, Beijing 100094, China; wanglixia20034@163.com

**Keywords:** ecological conservation redline (ECR), land use changes, CLUE-S model, ecosystem services value (ESV), landscape metrics

## Abstract

The Ecological Conservation Redline (ECR) of China plays an important role in avoiding ecological space occupancy and maintaining regional ecological security. Anji County in Zhejiang Province is one of the first regions to implement the ECR in China. This paper takes Anji County as an example to analyze the effects of ECR. To do this, we first set up two scenarios with the CLUE-S model: a normal land-use development scenario (NLDS) and an ECR implementation scenario (ECRS); then we compare the land use of 2010 and 2015 under NLDS and ECRS. Land use, ecosystem services value (ESV), landscape metrics, and ecological product outputs were compared between the entire county and the ECR areas. The results revealed the following: (1) From 2000 to 2015, the ecological land in Anji County decreased by 4.03%, while it decreased by 1.17% in the ECR areas. (2) In the ECR areas, there was less arable land and construction land of the ECRS than in the NLDS, which indicates the ECR impeded the expansion of construction land and arable land in the ECR areas. (3) The ECR areas account for 39% of Anji County but contribute more than 80% to the ESV of the whole county. During 2000–2015, the ESV of the entire county decreased while the ESV of the ECR areas increased. (4) From 2000 to 2015, whereas landscape fragmentation of the entire county increased, that of ECR areas decreased. (5) Since the ECR’s implementation, Anji County has vigorously developed the bamboo industry, ecological agriculture, the tourism industry, and achieved rapid economic development via industrial restructuring and transformation. On the whole, the ECR has neither adversely affected land development nor economic development but instead has promoted the optimization of the land’s spatial development pattern.

## 1. Introduction

With the development of industrialization and urbanization, most countries in the world confront prominent environmental problems, such as encroachment upon ecological space and continuous decline of ecosystem services, threatening the national and regional ecological security [1,2,3]. Protecting global key ecosystems, maintaining important ecological services, and conserving biodiversity become a priority. Delineation of protected areas (PAs) is regarded as an effective measure worldwide [4]. To date, over 15% of the earth’s land and 7% of its oceans has been designated as PAs [5]. China is one of the countries possessing rich ecosystem types and biodiversity. In order to protect its ecosystems, China establishes a national PAs system, which consists of three categories, named the natural protected areas, cultural protected areas, and comprehensive protected areas for nature and culture [6]. These PAs play important roles in protecting China’s natural ecosystems. However, at the same time, new problems arise related to PAs, such as spatial mismatch, isolation, conflicts between conservation and development, and so on [7]. The efficiency of PAs also needs to be improved, considering that approximately 30% of natural ecosystem types, 20% of wild animals, and 40% of higher plants are not included in natural reserves [1].

In response to such problems, the Chinese government has adopted a top-level project at the national level, called the Ecological Conservation Redline (ECR) [7,8,9]. It is considered an important innovation for delineating the insurmountable boundary of ecological security. Firstly, it integrates all areas needed to be conserved. On the basis of existing protection site networks, the most important ecological function areas, the important biodiversity areas as well as the ecologically fragile and sensitive areas are all integrated into ECR, which leads to more systematic and complete protection for ecosystems [10,11]. Secondly, the delineation of the ECR boundary is based on precise evaluation rather than experiences. In July 2017, the Ministry of Ecological and Environment of the People’s Republic of China (MEP) and the National Development and Reform Commission (MDRC) issued the guidelines for the delineation of ecological conservation redline. Many works of research also focus on the delineation of ECR. Frameworks, methods, and indices for assessing ecological function, ecological fragility, and biodiversity are widely investigated [12,13,14,15,16]. Thirdly, ECR highlights and guarantees strict management. Within the ECR areas, the land-use change and human activities will be strictly controlled. All activities should follow one common principle, that is, the area of ECR should not reduce, the ecosystem function of ECR should not decline and the ecological land of ECR should not covert to other types [17]. Since it was first proposed in 2011, ECR has attracted much attention from both researchers and government managers. By far it has evolved from theory to practice. 31 provinces have completed the delineation of ECR, among which the area of ECR can account for more than 50% of the area. However, whether ECR policy really works effectively in maintaining ecological security is a question that needs to be answered. Some people doubt the effects of such an ambitious project and worry that ECR may have negative impacts on socio-economic development [18]. Therefore, how to evaluate the effects of ECR has become a crucial issue [19].

Most previous research focused on the concept, significance, and delimitation of ECR in China [20,21,22,23]. Few studies assessed its effects. In spite of this, plenty of studies carried out on the effects of natural reserve regions [24] or large ecology project [25,26,27,28] can contribute to the evaluation of ECR. Firstly, land-use change, ecosystem service, and landscape metrics can be used as indicators to assess the impact of ecological protection policies. Secondly, the assessment of ECR should not be limited to the improvement of ecosystem functions, it may also include the evaluation of ECR’s impacts on regional economic and social development, which determine the overall effectiveness and success of the ecological protection policy. On the other hand, the majority of relevant quantitative studies failed to distinguish the role of the ecological protection policy and natural environment change, making it difficult to identify the contribution of policy to the effectiveness of ecological protection [29].

The ECR was only formally implemented nationwide in 2017, making it difficult to fully assess the effects of the policy’s implementation due to the relatively short time frame. However, it has been tested for a long time in some pilot places, which means case studies in the pre-pilot counties of the ECR can appropriately fill this gap. In this paper, we choose the Anji county, one of the earliest pilot places, as the study area, and construct an evaluation index system for the effect of ECR program implementation, covering four assessment items: land use, ecosystem service function, landscape pattern, and industrial economy development. Quantitative and qualitative methods are used to assess the effects of ECR in Anji county from time and spatial dimensions. The results of this paper are expected to clarify the usefulness of ECR for ecological protection, and therefore provide an important basis for further implementation of ECR in China.

## 2. Materials and Methods

### 2.1. Study Area

Anji County, in the northwest of Zhejiang Province, is located between 119°14′~119°53′ E and 30°23′~30°53′ N and covers an area of 1886 km^2^. At the end of 2015, the county had a registered human population of 464.1 million and the GDP was 30.33 billion yuan. The elevation is higher in the southwest and lower in the northeast. A subtropical oceanic monsoon climate characterizes Anji County, which is rich in water resources, including the source of the Taihu Lake and Huangpu River. This river system belongs to the Yangtze River system and consists of three drainage basins: Xitiaoxi, Dongtiaoxi, and Dongjin Rivers, which sustain 81 reservoirs, including two large reservoirs and three medium-sized reservoirs. Anji County has 71% forest coverage and abundant biological resources. In particular, it has six genera and 44 species of bamboo, earning the county the moniker “Bamboo Township of China”. Anji County is recognized across China for its white tea, chair industry, and bamboo flooring.

**Figure 1:**
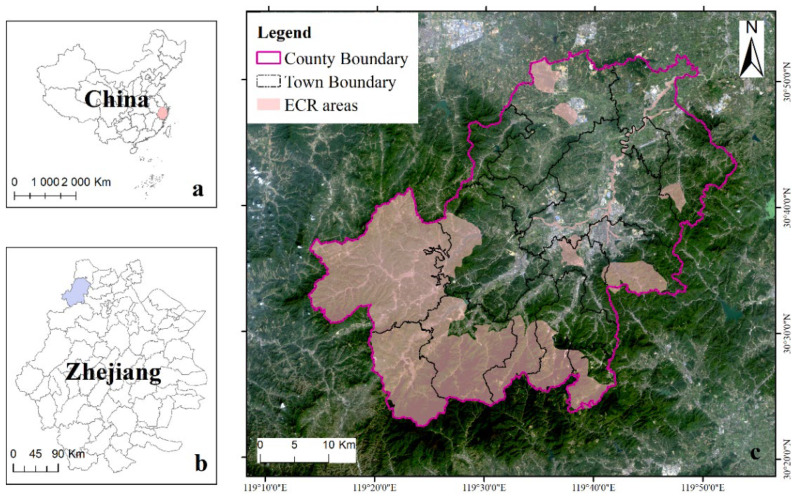
Location of Zhejiang Province in China (**a**), Anji County in Zhejiang Province (**b**) and the distribution of ECR in Anji county (**c**).

In 1998, Anji County abandoned the traditional path of industrial development, putting forward a development strategy in 2001 to become an ecological county. Around 2000, Chinese scholars proposed the ECR initiative and applied it in the *Anji Eco-County Construction Plan* [8]. They divided the critical ecological space into ECR areas, where strict protection was implemented, forming the rudiment of China’s ECR. The *Anji Eco-County Construction Plan* has been implemented since 2005, making the county one of the first regions in China to implement ECR. The ECR areas in Anji County is 736.48 km^2^, accounting for 39.02% of the county’s land area (Figure 1).

### 2.2. Methods

#### 2.2.1. CLUE-S Model for Land-Use Simulation

The CLUE-S (conversion of land use and its effects to a small region extent) model is developed on the basis of CLUE. It allows researchers to set the stability degree of different land-use types according to the historical changes of different land-use types in the land-use system and the actual situation of future land planning. The CLUE-S model can accurately simulate small-scale land-use changes, realize the synchronous simulation, and directly reflect the simulation results in the spatial location. It also has some limitations. For example, it can only simulate 13 land types at most, and there are restrictions on the number of enclaves and the number of grids in the study area. The CLUE-S model is similar to the ecosystem service value evaluation and landscape index based on land-use types, and there are many cases where the two methods are applied at the same time [30,31,32]. The CLUE-S model is used to stimulate the land-use pattern in the future based on the changes of the past and has been used in Europe, China, the Philippines, and many other regions [33].

The hypothesis of the CLUE-S model is that the land-use change of a region is driven by the land-use demand of the region, and the land-use distribution pattern of a region is always in a dynamic balance with the land demand and the natural environment and socio-economic conditions of the region. Based on the hypothesis of different types of land-use change, we can use the CLUE-S model to realize the synchronous simulation of different land-use changes.

The model consists of two independent core components, namely a non-spatial module and a spatial module. The non-spatial module calculates the total demand of land-use types. The data in the spatial module exists in the form of a grid, in which land use can be allocated according to different locations of the grid and transformed into land-use mode. Parameters such as restricted areas, land-use conversion elasticity, and transfer matrix need to be set in the space module. The test of the CLUE-S model is divided into two parts. Firstly, according to the results of logistic regression analysis, the interpretation ability of the driving factors can be tested by the method proposed by Pontius [34]. If the driving factors can well explain the land-use distribution pattern, the CLUE-S model can be used to continue the spatial allocation. Otherwise, the next spatial simulation cannot be carried out, and the driving factors with more explanatory power must be selected again. After space simulation, the Kappa index can be used to simulate the effect [35,36].

To analyze the impacts of the ECR on land-use changes, we designed two scenarios: a normal land-use development scenario (NLDS) and an Ecological Conservation Redline implementation scenario (ECRS). For the NLDS, the spatial land allocation in 2010 and 2015 was simulated under the precondition of land-use types in 2000 and 2005. For the ECRS, land use was the actual land-use situation after the implementation of the ECR in 2005.

In this research, the CLUE-S model was chosen to simulate land use under NLDS. To do this, the model was divided into two modules: one for the land-use demand module and one for spatial allocation. By analyzing the driving factors of land-use change—such as social economy, population, and policies and regulations—the land-use demand module calculates the yearly change in demand for different land-use types in the study area, and then distributes this demand in space, based on the spatial distribution module of raster data, to finally realize the simulation of spatiotemporal changes in land use. The spatial allocation module mainly reveals the relationship between the spatial distribution of land use and its driving factors, as well as spatial constraints, from which maps measuring the suitable degree of distribution of each land-use type in a given grid unit were generated. In the CLUE-S model, according to the land-use pattern and relevant driving factors, logistic stepwise regression was used to diagnose the probability of a certain land-use type appearing in each grid. In this paper, distance to rivers, distance to roads, population density, elevation, and slope were selected as driving factors of land-use changes.

The conditional probability of certain land-use types distributed in a grid is:

$$P_{i}=P\left(y_{i}=1/x_{i}\right)$$

This probability can be expressed in the following logistic function form:

$$P_{i}=\frac{exp\left(\beta_{0}+\beta_{1}X_{1i}+\beta_{2}X_{2i}+\cdots +\beta_{n}\beta_{ni}\right)}{1+exp\left(\beta_{0}+\beta_{1}X_{1i}+\beta_{2}X_{2i}+\cdots +\beta_{n}\beta_{ni}\right)}$$

where, *P_i_* indicates the probability that each grid may appear a certain land-use type *i*; *X*_1*i*_, *X*_2*i*_, …, *X_ni_* indicate the driving factors of land-use type *i*; *β*_0_ is a constant term, *β*_1_, *β*_2_, …, *β_n_* are the regression coefficients of the explanatory variable *X_k_* (*k* = 1, 2, …, *n*). A linear function can be obtained by transforming the formula.

$$Log\left(\frac{P_{i}}{1-P_{i}}\right)=\beta_{0}+\beta_{1}X_{1i}+\beta_{2}X_{2i}+\cdots +\beta_{n}\beta_{ni}$$

The simulation effect of the CLUE-S model was quantitatively evaluated by the Kappa index. This index is used primarily to evaluate the accuracy of a classification image. Hence, it can be used to objectively evaluate the simulation effect of the CLUE-S model. It is expressed this way:

$$Kappa=\frac{P_{0}-P_{C}}{P_{p}-P_{c}}$$

where, *P*_0_ is the proportion of the correct simulation; *P_c_* is the proportion of the expected simulation in random cases; *P_p_* is the proportion of the correct simulation in ideal classification cases. Thus, the closer the Kappa index is to 1, the better the simulation results are. For Kappa index values between 0.41 and 0.60, model simulation consistency may be interpreted as a medium; values between 0.61 and 0.80 would indicate a model simulation consistency that is good.

#### 2.2.2. Ecosystem Services Value Evaluation

On the basis of Costanza’s assessment of global ecological assets [37], Zhang worked out the equivalent factor table of ecosystem service value (*ESV*) for China—pointing out that an ecosystem’s services are closely related to its biomass—by revising the biomass parameters to reflect the regional differences of *ESV* [38]. That method was widely adopted and cited by others [39]. So, using the equivalent *ESV* data for China and referring to the adjustment method of *ESV* coefficients proposed by relevant researchers [40], this paper uses two parameters, net primary productivity (*NPP*) and normalized difference vegetation index (*NDVI*) to revise the *ESV*. Doing so enabled us to obtain more accurate evaluation results for *ESV*. This formula was used:

$$ESV=\sum \left(A_{k}\times VC_{k}\right)$$

where, *ESV* is the ecosystem services value; *A_k_* is the area of land-use type *k*; *VC_k_* is the unit area ecosystem service value of *k*: *k* can be forest, grassland, arable land, or water bodies.

The formula for calculating the adjustment coefficient of *ESV*:

$$R_{i}=\left[\frac{NPP_{i}}{NPP_{mean}}+\frac{f_{i}}{f_{mean}}\right]/2$$

where, *NPP_mean_* and *f_mean_* are, respectively, the mean values of *NPP* and vegetation coverage (*f*) of the ecosystem in the region; *NPP_i_* and *f_i_* are the *NPP* and vegetation coverage (*f*) in the *i* pixel. Vegetation coverage (*f*) is calculated by *NDVI* in this way:

$$f=\frac{NDVI-NDVI_{min}}{NDVI_{max}-NDVI_{min}}$$

#### 2.2.3. Landscape Metrics

Landscape metrics refer to a series of quantitative indexes that can accurately describe the characteristics of landscape composition and spatial distribution; hence, they can convey much information about the landscape pattern [41]. Fragstats v4.2 is a powerful software tool for calculating landscape metrics: it can calculate more than 100 indicators of landscape patterns at the patch level, the type level, and the landscape level. Indexes reflecting landscape fragmentation, connectivity, and heterogeneity, namely the largest patch index (LPI), edge density (ED), landscape shape index (LSI), Shannon’s diversity index (SHDI), aggregation index (AI), and contagion index (CONTAG), were all selected to study the landscape pattern changes of the ECR areas and the entire Anji County [42].

### 2.3. Data

The respective land-use data with a spatial resolution of 30 m of Anji County, in 2000, 2005, 2010, and 2015, were obtained from the National Ecological Environment Remote Sensing Survey Database, based on multi-sources remote sensing data. Both the NPP and NDVI data came from the United States Geological Survey (https://www.usgs.gov/, (accessed on 20 June 2019), with a spatial resolution of 250 m and a temporal resolution of 16 days. After transformation of the projection coordinate system, spatial resampling, and other data processing steps, the spatial resolution data consistent with land use can be obtained. The data sets for the terrain, road network, and river system came from the National Earth System Science Data Sharing Service Platform (http://www.geodata.cn/index.html, accessed on 20 June 2019). Economic and social statistics were collected from the statistical yearbooks of Zhejiang Province, Huzhou City, and Anji County, from 2000 to 2015.

## 3. Results

### 3.1. Land-Use Change

**Figure 2:**
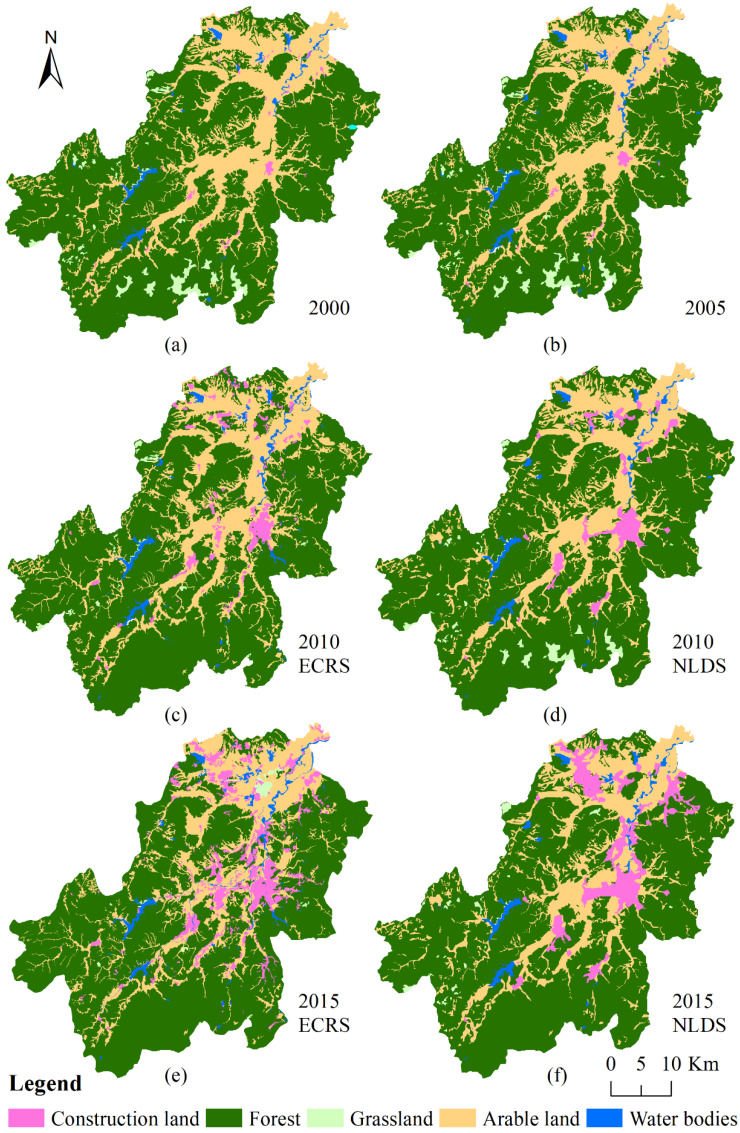
Land use of 2000 (**a**), 2005 (**b**), 2010 and 2015 in Anji County under an Ecological Conservation Redline implementation scenario (ECRS) (**c**,**e**) and a normal land-use development scenario (NLDS) (**d**,**f**).

Taking 2000 as the base year, and 2005 as the simulation target year, the simulation results were compared with the actual land-use distribution (Figure 2), and the simulation accuracy was then verified. The Kappa coefficient was 0.86, which suggested a very good model simulation effect. Then, taking 2005 as the base year and 2010 and 2015 as the target year, respectively, the land-use change was simulated.

To better compare the effectiveness of ECR, we classified forest, grassland, water bodies, and unused land as “ecological land” as referred to in the previous research [43]; we then analyzed the respective changes over time in coverage of ecological, arable, and construction land.

As shown in Table 1, from 2000 to 2015, the ecological land decreased by 4.03% in the entire county, while it only decreased by 1.17% in ECR areas, which means the ECR protects the ecological land effectively. If ECR is not implemented, the ecological land would decrease by 1.66% in ECR areas under NLDS.

Considering land-use change slightly and nearly maintains the same trend both in ECR areas and the entire county before 2005 (Table 1), we can assume that there are no intrinsic differences between the two regions. After implementing ECR, the construction land of 2015 in the entire county under NLDS was less than ECRS, but the situation was just the opposite in the ECR areas. In 2015, there was less arable land and construction land in the ECRS than in the NLDS in the ECR areas, which indicates the ECR impeded the expansion of construction land and arable land in the ECR areas.

### 3.2. Ecosystem Services Value Changes

In 2015, the ECR areas account for 39.02% of Anji County but contribute more than 84.38% to the ESV of the whole county. During 2000–2015, the ESV of the entire county decreased slightly, with an average rate of 1.01 million yuan a year. At the same time, a fluctuating increasing trend of ESV within the ECR areas was evident, with an average rate of 0.77 million yuan a year (Table 2). Especially after implementing the ECR in 2005, ESV within the ECR areas increased from 2520.66 to 2568.92 million yuan in 2015, and the proportion of ESV_ECR_ rose from 82.21% to 84.38% in the same period. This indicates that ECR has played an important role in protecting important ecosystems and maintaining ecosystem services in Anji County.

Many previous studies tend to analyze the changes in ecosystem services and consider improving ecosystem services the main index by which to measure the benefits of ecological protection [16,44]; most of the research shows that ecological protection figures prominently in enhancing regional ecosystem services [45]. This research is undoubtedly in line with the previous research on ecological protection effectiveness analysis.

### 3.3. Landscape Pattern Changes

The landscape metrics across Anji County and for its ECR area were calculated. As shown in Table 3, LPI, AI, and CONTAG of the entire county decreased from 2000 to 2015, while its ED, LSI, and SHDI all increased. This indicated that landscape fragmentation was accelerating and landscape connectivity was declining, and that heterogeneity had increased overall. For the same period, the LPI, AI, and CONTAG of the ECR areas surpassed those of the entire county whereas its ED, LSI, and SHDI were lower; hence, there was greater landscape connectivity in the ECR area. After the ECR’s implementation in 2005, the LPI and CONTAG of the ECR areas shifted, going from a slight decline to a significant increase, and its AI continued to rise, while its ED, LSI, and SHDI all had a downward trend. So, the changes in landscape metrics for the ECR areas followed a trend contrary to those measured at the whole-county level. The implementation of the ECR has played an active role in stabilizing the landscape pattern and protecting regional ecosystems.

Considering the landscape changes of the entire county and those of the ECR areas, the implementation of ECR, on the one hand, has reduced fragmentation of the landscape within the ECR areas; on the other hand, fragmentation of the landscape outside the ECR areas has obviously increased. This suggests that the ECR has exerted significant effects on the integrity and connectivity of the ecosystems in the protected area, yet, at the same time, development activities will continue outside the ECR areas. So, implementation of ECR will promote overall optimization of the protection and development pattern of territorial space.

### 3.4. Industrial Economy Development

From 2005 to 2015, the total GDP of Anji County increased more than three-fold, from 8.852 to 30.304 billion yuan, at an average annual growth rate of 12.67%, a value exceeding that of Zhejiang Province and the whole country over the same period. During this time, Anji County strove to develop an ecological industry (bamboo forest industry), ecological agriculture, and the tourism industry, and has achieved rapid economic development through industrial restructuring and transformation. Research has found that intensifying management can increase the economic benefits of the bamboo forest industry, which not only brings additional carbon benefits but also substantial gains in timber and shoots as co-benefits [46].

As a result of the changes in tourism income, its proportion of the GDP has risen from 10.74% in 2005 to 57.21% in 2015. Tourism resources in Anji County are mainly distributed inside the ECR areas and the surrounding vicinity. Relying on its mountain landscape, Anji County has established scenic sites, such as Longwangshan Natural Exploration Park, the Tianjia Mountain Insect Record Paradise, Dashilang Scenic Area, and the North Zhejiang Grand Canyon. Relying on its main water bodies, namely the Huxi River, Longwangxi River, Xixi River, Dipu River, and Nanxi River, Anji County has also built the Anji Natural Traceability Park and developed and constructed several hydrophilic tourism projects, such as the Laoshikan Reservoir, Phoenix Reservoir, Sanguan Wetland, and Xiwei River. Relying on its pastoral landscape, Anji County has constructed four distinctive pastures: Shao Wu Five-color Cultural Pastoral, Bijiashan Shuxiang Pastoral, Shangshuyuan Traditional Chinese Studies Pastoral, and the Xilong Huangdu Wanmu Tea Garden. Finally, relying on its vegetation, Anji County has implemented plant sightseeing projects, such as the China Bamboo Ocean, Bamboo Expo Park, and Central South Hundred Grass Garden.

Further, Anji County has focused on tapping other biological resources, to produce characteristic ecological products and to create a mature industrial development model. Anji white tea, bamboo, bamboo shoots, silkworms, and other agricultural products now enjoy a high-quality reputation. The chair industry and bamboo industry, as well as green textiles, biomedicine, equipment manufacturing, new energy and new materials, green foods, and other industries, all benefit from having ecological friendly characteristics. Among them, the processing of bamboo products has realized the efficient utilization of bamboo, from leaf to root parts, from physics to chemistry, and thereby laid a sound foundation for developing a local circular value-added economy. According to the output statistics of Anji County’s main ecological products (Table 4), the production of bamboo shoots, Chinese chestnuts, bamboo, and miscellaneous bamboo in the ECR areas accounted for 40%–50% of the total output of the county.

## 4. Discussion

ECR could provide effective ecosystem management over China’s vast geographic area [1,11]. The Chinese government implemented the designation of ECR on the national scale in 2017 and established an overall ECR system nationwide in 2020 [17]. The ECR policy is designed to constrain human activities in areas that are maintaining national ecological security or providing essential ecosystem services. From the perspective of environmental management, they aim to define regions with unique and important ecological roles. Compared to PAs, ECR further expands its scope of protection by including areas of high ecological importance such as water and soil conservation, as well as ecologically sensitive and vulnerable areas. ECR establishes a unified supervision system on the national scale to make management more efficient. By incorporating ECR management into local government assessments, ECR further ensures the rigor of ecological management [47]. In this paper, we construct an evaluation index system for the effect of ECR program implementation, covering four assessment items: land use, ecosystem service function, landscape pattern, and industrial economy development. It evaluates ecosystem quality in terms of composition, pattern, function, and services and also evaluates socio-economic effects. Our assessment in Anji county proves the effectiveness of the ECR program, consistent with the view of most academics.

In addition, restricted by ground-collected data, this paper used remote sensing survey and spatial data, at the county scale, primarily to reveal the role of the ECR in maintaining ecosystem services. In order to further clarify the benefits of the ECR, it is necessary to conduct in-depth research on the processes and mechanisms that maintain regional ecosystem services and their external benefits, based on the relationship between ecosystem and human well-being. Looking ahead, firstly, observation experiments are needed to study the scope and process of ecosystem services’ generation and transfer, as well as the mechanisms of pollutant removal in a given ecosystem, to scientifically evaluate the ecological and environmental benefits of the ECR. Secondly, it is necessary to follow the supply, trade, and consumption of ecological products to analyze the full scope of economic benefits provided by ecosystems. Thirdly, we should link ecological protection with the county residents’ well-being, establish feedback and linkages, and clarify the main characterization of ecosystem protection and well-being. Finally, it is necessary to study the external benefits of the ECR and to explore its comprehensive benefits by identifying the sources and links of different ecological resources.

## 5. Conclusions

This paper takes Anji County as a case study area to compare changes in land use and ESV for the entire county and for its ECR areas, under the NLDS and the ECRS, respectively, to evaluate the role and impact of the ECR on ecological protection and economic development. The main conclusions drawn are as follows:

(1) From the perspective of land-use change, the ECR has not affected the demand for land development and utilization in the county; on the contrary, it has done more to safeguard the ecological land of important ecological regions and promoted the optimization of the regional land development pattern. At the same time, under the ECRS, there is less construction land and arable land available than under the NLDS, but there is more ecological land maintained. This proves that the ECR, to a certain extent, has prevented the ecological land from being occupied within ECR areas, so that ecosystem services and functioning are better maintained.
(2) The ESV was stable inside the ECR areas in Anji County, at approximately 2.5 billion yuan, from 2000 to 2015. The proportion of ESV provided by the ECR areas accounts for more than 80% of the county’s total value. Moreover, the ESV of the ECR areas has increased continuously while the ESV of the whole county has declined. Hence, ECR is crucial for protecting important ecosystems and maintaining their services in the county.
(3) From 2000 to 2015, landscape fragmentation increased, connectivity decreased, and heterogeneity increased in Anji County. By contrast, landscape fragmentation has generally decreased within the ECR areas, especially after 2005. This confirms that ECR helps to ensure the integrity and connectivity of the county’s ecological land.
(4) Under the ECR policy, Anji County has developed a thriving ecological industry e.g., the bamboo forest industry, ecological agriculture, and tourism industry, and has achieved rapid economic development via industrial restructuring and transformation. Ecosystems in ECR provide more high-quality ecological resources for economic development. Correspondingly, ecosystems are constantly transformed into invaluable assets and become the source of the county’s productivity and competitiveness. This indicates that we can have a win-win goal, that is, promoting development through protection and strengthening protection through development.

## Figures and Tables

**Table 1:** Anji County land use results under ECRS and NLDS (km^2^).

Region	Land-Use Type	Base		ECRS		NLDS	
		2000	2005	2010	2015	2010	2015
Entire county	Construction land	8.72	10.98	52.91	134.05	55.63	126.55
	Arable land	476.98	475.68	465.97	410.46	444.69	411.3
	Ecological land	1402.57	1401.33	1367.73	1346.02	1384.64	1347.11
ECR areas	Construction land	1.53	1.70	6.99	13.68	5.30	14.67
	Arable land	80.14	77.97	79.03	71.73	78.6	76.22
	Ecological land	655.03	657.02	649.04	647.38	651.14	644.15

Note: NLDS represents a normal land-use development scenario, and ECRS represents an Ecological Conservation Redline implementation scenario.

**Table 2:** Ecosystem services value (ESV) of the entire county and the Ecological Conservation Redline (ECR) areas.

	2000	2005	2010	2015
ESV_ANJI_ (million yuan)	3059.64	3066.30	3045.60	3044.47
ESV_ECR_ (million yuan)	2557.39	2520.66	2576.63	2568.92
Proportion of ESV_ECR_ (%)	83.58	82.21	84.60	84.38
Density of ESV_ANJI_ (million yuan/km^2^)	1.62	1.62	1.61	1.61
Density of ESV_ECR_ (million yuan/km^2^)	3.47	3.42	3.51	3.51

Note: ESV_ECR_ represents the ESV of the ECR areas, and ESV_ANJI_ represents the ESV of the entire county. Proportion of ESV_ECR_ was calculated as ESV_ECR_/ESV_ANJI_. Density of ESV_ANJI_ was calculated as ESV_ANJI_/area of Anji County. Density of ESV_ECR_ was calculated as ESV_ECR_/area of the ECR areas.

**Table 3:** Landscape metrics changes of the ECR areas and for the entire Anji County.

Landscape Metrics	2000	2005	2010	2015
LPI_ANJI_	68.14	67.09	66.68	66.51
LPI_ECR_	70.39	70.04	76.67	77.44
ED_ANJI_	13.01	13.05	14.00	15.19
ED_ECR_	10.78	10.75	9.76	9.91
LSI_ANJI_	16.11	16.17	17.23	18.42
LSI_ECR_	11.98	11.99	11.35	11.34
CONTAG_ANJI_	61.79	61.07	59.16	54.15
CONTAG_ECR_	66.81	66.38	70.11	70.35
SHDI_ANJI_	0.74	0.76	0.79	0.88
SHDI_ECR_	0.64	0.65	0.57	0.56
AI_ANJI_	83.33	83.28	82.06	80.67
AI_ECR_	83.34	83.36	84.52	84.41

Note: LPI_ECR_ represents the LPI of the red line area of ecological protection, and LPI_ANJI_ represents the LPI of the entire county. Other landscape metrics have similar subscript meanings.

**Table 4:** Statistics of main ecological products output, in 2015, from Anji County and from its Ecological Conservation Redline (ECR) areas.

	Bamboo Shoots (t)	Chinese Chestnuts (t)	Mao Bamboo (Million)	Miscellaneous Bamboo (t)
Entire county	6750	2950	29.70	38500
ECR areas	3290	1259	13.79	18050
Proportion (%)	48.74	42.68	46.43	46.88
